# Comparison of synergy patterns between the right and left hand while performing postures and object grasps

**DOI:** 10.1038/s41598-023-47620-9

**Published:** 2023-11-20

**Authors:** Prajwal Shenoy, Anurag Gupta, Varadhan S.K.M.

**Affiliations:** 1 Department of Mechatronics Engineering, Manipal Institute of Technology, Manipal Academy of Higher Education, Manipal, Karnataka 576104 India; 2 Department of Applied Mechanics and Biomedical Engineering, Indian Institute of Technology Madras, Chennai, Tamil Nadu 600036 India

**Keywords:** Biomedical engineering, Motor control

## Abstract

The human hand, with many degrees of freedom, serves as an excellent tool for dexterous manipulation. Previous research has demonstrated that there exists a lower-dimensional subspace that synergistically controls the full hand kinematics. The elements of this subspace, also called synergies, have been viewed as the strategy developed by the CNS in the control of finger movements. Considering that the control of fingers is lateralized to the contralateral hemisphere, how the synergies differ for the control of the dominant and the non-dominant hand has not been widely addressed. In this paper, hand kinematics was recorded using electromagnetic tracking system sensors as participants made various postures and object grasps with their dominant hand and non-dominant hand separately. Synergies that explain 90% of variance in data of both hands were analyzed for similarity at the individual level as well as at the population level. The results showed no differences in synergies between the hands at both these levels. PC scores and cross-reconstruction errors were analyzed to further support the prevalence of similarity between the synergies of the hands. Future work is proposed, and implications of the results to the treatment and diagnosis of neuromotor disorders are discussed.

## Introduction

Owing to the direct monosynaptic connections from the motor cortex to the alpha-motor neurons innervating the hand muscles, and the disproportionately large representation of the fingers in the motor cortex, the dexterity and control of the human hand is unmatched. This dexterity and control allows humans to manipulate objects having complicated contours with ease as well as allows for the performance of a large number of hand postures like those performed in American Sign Language [ASL]. With the hand having a large number of joints, how the brain controls such a redundant manipulator has intrigued researchers for many decades. Studies have shown that the kinematics of the human hand can be represented in a lower dimensional latent space, wherein a few control units called synergies (or eigen postures) can be used to represent the kinematics of the entire hand^1–5^. Through principal component analysis (PCA), it has been found that a larger variance in finger movements (around 70%-90%) can be explained by as less as the first three synergies^1,3,5^. Hence, these three synergies contain most of the postural and kinematic information. Synergy based studies have hypothesized that instead of the CNS controlling each joint of the hand individually (which is a computationally daunting task), it controls the weights of these synergies to generate hand movements. A linear combination of these weighted synergies results in the desired hand posture.

A body of literature has emerged that has linked synergies to motor areas of the brain. For example, EEG recordings have been used to decode kinematic synergies^6,7^, neuronal activity in the primary motor cortex determined through fMRI images have been used to predict hand postural synergies^8^, finger movements evoked by stimulating the motor areas in the cortex (using TMS) and finger movements due to voluntary control have been found to have similar postural synergies^9^, etc. These studies provide evidence for correlation between hand synergies and activity in the cortical motor areas. Hence, they lend support to the hypothesis that the CNS controls hand movements by tuning the weights of synergies. Keeping these points in mind, investigating synergies could perhaps give insights into the strategies employed by the CNS in controlling hand movements. Apart from this, synergy-based research has also aided in the development of better robotic hands^10,11^ and in disease diagnosis and treatment^12^.

An area in this field that has not been thoroughly investigated is the comparison between the synergies (i.e., the lower dimensional latent space representation of hand movements) of the right-hand (RH) and the left-hand (LH). We hypothesize that the RH and LH synergies will be different. This hypothesis is based on several reasons, the most obvious being the contrasting way in which the dominant (DOM)/preferred hand is used in comparison to the non-dominant (NDOM) hand in everyday activities. This contrast in usage could perhaps be a manifestation of the differences in control strategies/commands (and in turn synergies) used by the CNS for the control of the DOM and NDOM hands. Another critical reason is the asymmetries that exist in the structure and function between the motor areas of the right and left cerebral hemispheres^13^. Several studies provide evidence to support this. For e.g., a study showed that the depth of the central sulcus (also an indicator of the area that represents hand movements) is greater in the hemisphere contralateral to the dominant hand^14^. A magnetoencephalography (MEG) based study showed that the volume of the primary motor cortex that represents certain hand movements was larger in the hemisphere contralateral to the DOM hand when compared to the volume contralateral to the NDOM hand^15^. A transcranial magnetic stimulation (TMS) based study showed that certain excitatory and inhibitory mechanisms in the primary motor cortex were more pronounced in the DOM than the NDOM hemisphere for a group of right-handers^16^. Finally, a study provided evidence for the control of complex movements being lateralized to the left hemisphere^17^. These examples are just a subset of a larger group of studies that provide evidence for asymmetries in motor areas between the cerebral hemispheres. Apart from this, the output of motor commands for the hands is lateralized to the contralateral hemisphere (for e.g., stimulating the hand region of the left motor cortex will result in movement of the contralateral right hand^18^). In this regard, it would not be unreasonable to expect that these asymmetries might result in different movement strategies (and in turn different synergies) being lateralized to the DOM and NDOM hemispheres. This in turn probably manifests as the manual asymmetries seen in the hands.

Only a few studies have investigated the postural and kinematic synergies of the DOM and NDOM hands^5,19–21^. These studies have computed synergies by arranging the data in three different ways: DOM hand and NDOM hand data of all participants concatenated vertically^5^, DOM hand and NDOM hand data concatenated horizontally (also called bimanual synergies)^19,20^, DOM hand and NDOM hand data analyzed separately^21^.

In the first study^5^, the data set consisted of static hand postures during object grasps. Since the dataset was concatenated vertically, an explicit comparison of synergies between the RH and LH was not possible. Instead, the PC scores (obtained by projecting data onto the synergies) were computed for the DOM and NDOM hands and compared. The analysis revealed significant differences between the PC scores of the DOM and NDOM hand for the first and fourth synergy. However, no interpretation or discussion was provided to elucidate the reason for these differences. Furthermore, the analysis was performed on static hand postures. Analysis of dynamic hand movements could perhaps provide more insights with regard to the differences between the DOM and NDOM hand synergies. In the studies involving bimanual synergies, the authors put forward the possibility of a simpler and simultaneous control of the DOM and NDOM hands^19^ as well as arms^20^. They proposed that the CNS controls movements of both the upper limbs through a single synergy rather than controlling them via two separate synergies. While, such a bimanual control could greatly simplify robotic arm designs, its applicability to study the control strategies employed by the CNS for the control of the DOM and NDOM hands is limited since research in neuroscience has provided evidence for the control of distal parts of the upper limb being lateralized to the contralateral cerebral hemisphere^18^ i.e., two separate locations for the generation of control commands for the DOM and NDOM hands respectively. Keeping these points in mind, analyzing the LH and RH synergies separately would give better insights into the control strategies employed by these two separate regions of the CNS. Finally, a recent study on the muscular synergies of the LH and RH found no evidence for differences in the synergistic activation patterns across limbs but differences only at the population level^21^. The analysis was performed on the EMG data recorded while participants performed ASL postures with the RH and LH separately in two different sessions.

While studies on the dynamic dominance hypothesis with regard to the upper limbs have shown clear differences in the dynamics between the DOM and NDOM limbs and their roles in everyday behaviour^22,23^, whether such differences exist in the kinematics space of distal regions like hands and fingers still remains unclear. Hence, considering the limitations in the above studies, we attempt to compare the synergy patterns of the RH and the LH separately as participants perform various postures and object grasps, and check if similar results as demonstrated in the muscle synergy space^21^ are observed in the kinematics space as well. The resulting synergies of the RH and LH will be compared across the limbs of individuals as well as at the population level. A cross-reconstruction will be employed to check if the lower dimensional representation of one hand can be used interchangeably to reconstruct the postures of the other hand. Implications to the treatment and diagnosis of neuromotor disorders from the analysis of the LH and RH synergies will be discussed.

## Materials and methods

### Participants

Ten right-handed participants, five males and five females aged 29 ± 3.6 (mean ± S.D) participated in the study. The participants provided written informed consent and had no history of neuromotor disorders and injury to hands and/or arms. The handedness of the participants was confirmed using the Edinburgh handedness inventory score. The experimental procedures were approved by the Institute Ethics Committee (IEC) of IIT Madras (IEC/2020–03/SKM/02/10). All the experimental sessions were performed in accordance with the procedures approved by the Institute Ethics Committee of the Indian Institute of Technology Madras.

### Data collection

**Figure 1:**
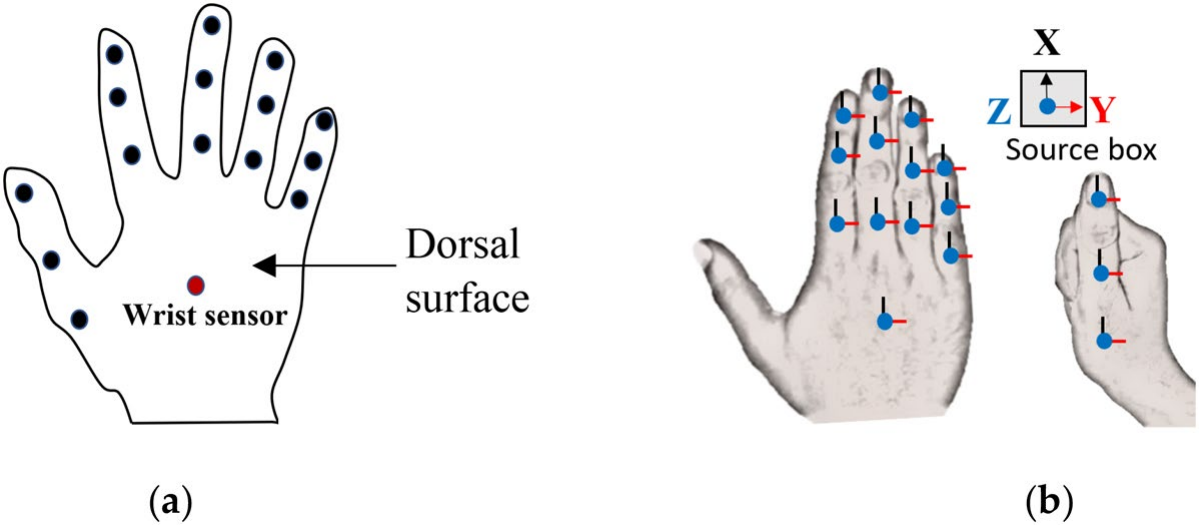
Details of the data collection process (**a**) Placement locations of the EMTS sensors on the dorsal surface of the hand. The sensor on the wrist is used as the reference sensor. (**b**) The EMTS sensors reference frames post the sensor-to-segment alignment.

Quaternion orientation data of all the finger phalanges and wrist was recorded using the Electro Magnetic Tracking System (EMTS)—Polhemus Liberty™ 240/16 (static accuracy 0.15° for sensor orientation). For this purpose, 16 EMTS sensors (model: Polhemus Micro Sensor 1.8™) were mounted on the dorsal surface of the hand, as shown in Fig. 1a. An EMTS source box was kept close to the hand and served as the global reference frame for all the EMTS sensors. A two-surface adhesive tape was used below the EMTS sensors, and surgical tape was used on top to firmly attach the EMTS sensors to the hand. Quaternion data was collected at an update rate of 100 Hz using code developed in the LabVIEW software.

### Sensor-to-segment alignment

It is practically not possible to perfectly align the EMTS sensors to the finger phalanges, hence the orientation of the sensors does not accurately represent the orientation of the phalanges. In order to overcome this problem, the sensor reference frame needs to be aligned to the finger phalange it is attached to. This is called sensor-to-segment alignment and is done in two steps in our experiment. (1.) In the first step, the wrist and all the fingers (except the thumb) are kept flat on the table and aligned along the positive x-axis of the EMTS source box, as shown in Fig. 1b. Then a command called “boresight” is executed using the Polhemus system. This command, via software, rotates the local reference frames of the EMTS sensors to align with the global reference frame of the EMTS source box i.e., even though the sensors are not physically aligned to the global reference frame, the local reference frames that represent these sensors are aligned to the global reference frame via software. Now, the local reference frames of the sensors represent the orientation of the finger phalanges to which they are attached to as the fingers were aligned to the global reference frame at the time of the boresight command execution. (2.) In the second step, the process remains the same, the only difference being that the thumb is aligned to the global reference frame instead of the four fingers and the wrist. A more detailed explanation of this entire process is given in the following reference^24^.

### Experimental protocol

**Figure 2:**
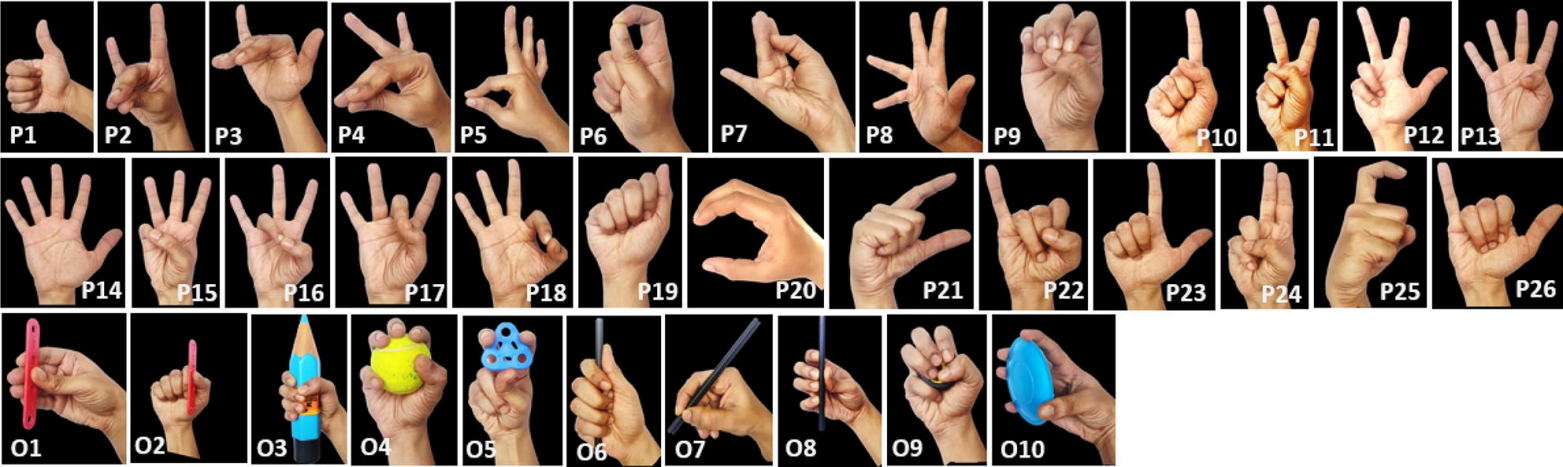
Static phase of the 36 movements of the experiment are depicted here. P1 to P9 depicts eight Bharatnatyam postures, P10 to P19 depicts ten ASL numbers, and P20 to P26 depicts eight ASL letters. O1 to O10 depict ten object grasps that cover a wide variety of grasp taxonomies.

Each participant was instructed to perform 36 different hand movements. These movements consisted of the generation of 26 different hand postures and the grasping of 10 different objects (See Fig. 2). The hand postures comprised of postures from the Bharatanatyam dance (a classical Indian dance form), ASL letters, and ASL numbers. The object grasps included grasping a variety of objects to replicate commonly used grasps such as pinch grasp, power grasp, tripod grasp, and palmar grasp. For each participant, the experiment was conducted across two sessions which were spaced not more than 10 days apart. In the first session, the 36 movements just mentioned were performed with the RH, and in the second session, the same 36 movements were performed with the LH. For each of the 36 movements, the participant performed three trials (36 × 3 = 108 trials). The duration of a trial was 8 s. For every trial, a picture of the movement to be performed was displayed to the participant via a monitor. At the start of every trial, the participant was instructed to keep the hand flat on a table with all the fingers adducted. We refer to this as the home position. 1 s into the trial, a verbal cue was given to the participant to begin performing the movement. The participant had to make the posture or grasp the object and then keep the hand stationary at that posture/object grasp for a duration of approximately 4 s. At around the 6 s mark, another verbal cue was given to the participant to move the hand back to its initial home position. The trial ended at the 8th s. Before the start of the experiment, a practice session was conducted where the participants were shown images of all 36 movements and were asked to practice them a few times. (Note: A part of the collected data set (RH session of 5 participants) has been used in another study^25^).

### Preliminary data processing

As a first step in data analysis, the relative quaternion across the two adjacent segments of a joint was computed using (1). This was done for all the joints.

1 $$q_{YrelativeX} = q_{X}^{conj} \otimes q_{Y}$$

Here, $q_{{\text{X}}}$ and $q_{Y}$ represent the orientation of two adjacent segments X and Y of a joint, respectively (where X is the proximal segment, and Y is the distal segment), $q_{X}^{conj}$ is the conjugate of $q_{X}$ and $q_{YrelativeX}$ represents the orientation of segment Y relative to segment X. $\otimes$ represents quaternion multiplication. For the purposes of animation and posture reconstruction of a 3D hand model, the relative quaternions between each segment were converted to their corresponding Euler angles with “X, Z, Y” (roll, yaw, pitch) as the intrinsic rotation sequence.

For performing dimensionality reduction using Principal Component Analysis (PCA), the direct application of linear methods on quaternions is not valid since quaternions are defined on a manifold that is non-linear. Hence, logarithmic mapping was first performed to linearize the quaternions. PCA was then performed, and further analysis was done. Finally, exponential mapping was performed to convert the data in linear form back to quaternions. A similar approach has been previously used in^24–26^. A brief explanation of the logarithmic maps and exponential maps is provided as supplementary material.

### Dimensionality reduction using PCA

**Figure 3:**
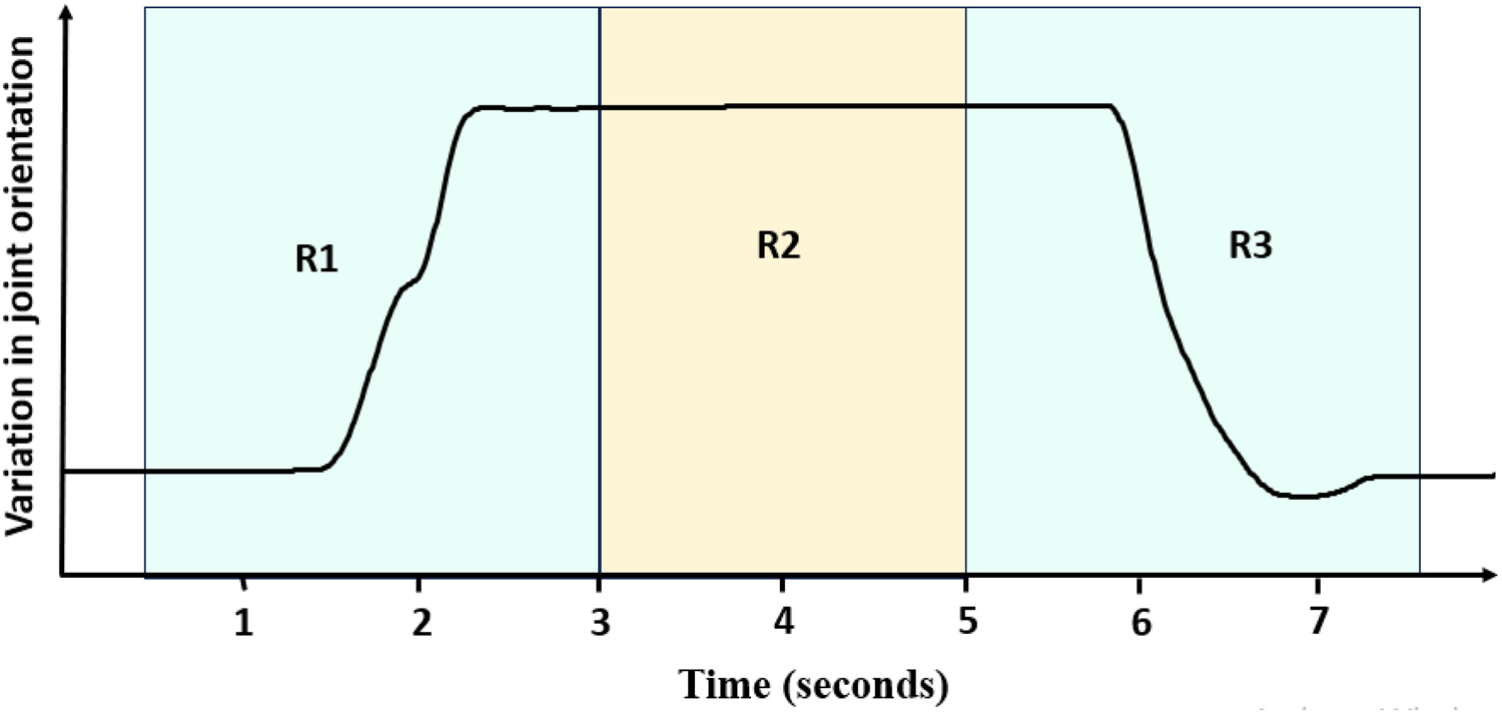
Data splitting for analysis. Analysis of synergies is performed for 3 conditions: dynamic data, static data, and full set data. A single trial data of 8 s is split into three regions R1 (0.5 s to 3 s), R2 (3 s to 5 s), and R3 (5 s to 7.5 s). Regions R1 and R3 are concatenated to form the dataset for dynamic data analysis. Region R2 forms the dataset for static data analysis. Data of the entire 8 s is used for full set data analysis.

To compare the synergies between the LH and RH, PCA was employed. The dataset for each participant comprised of 2 matrices, one each for the RH and LH. Each “hand matrix” consisted of relative quaternions of 15 joints recorded while performing 36 movements. Thus, each matrix had a size of 86,400 rows (36 (movements) × 3 (trials per movement) × 100 (sampling frequency) x 8 s (trial duration)) and 60 columns (4 (1 quaternion = 4D number) × 15 (joints)). This data matrix is called as the full set data matrix. However, the two “hand matrices”/ “full set data matrices” could not be directly compared since similar movements of the LH and RH are essentially mirror movements of each other (for e.g., abduction movement of the LH and RH are in opposite directions). In order to compare the two matrices, appropriate transformations were made to the relative quaternions of the LH matrix such that movements represented by this transformed LH matrix were mirror movements to the original LH matrix. All further analysis was done on the transformed LH matrix and the RH matrix. The full set data matrix for each hand was further divided into 2 more matrices. The first matrix comprised of joint orientations in regions R1 and R3 (See Fig. 3) combined and is called the dynamic data. The second matrix comprised of joint orientations from the region R2 (See Fig. 3) and is called the static data. The dynamic data matrix included orientations from 0.5 s to 3 s (2.5 s of data corresponding to region R1) and data from 5 s to 7.5 s (2.5 s of data corresponding to region R3). Thus, the dynamic data matrix had a size of 54,000 rows (36 (movements) × 3 (trials per movement) × 100 (sampling frequency) x 5 s (trial duration)) and 60 columns (4 (1 quaternion = 4D number) × 15 (joints)). The static data matrix had a size of 21,600 rows (36 (movements) × 3 (trials per movement) × 100 (sampling frequency) x 2 s (trial duration)) and 60 columns (4 (1 quaternion = 4D number) × 15 (joints)). In each category, the RH data of the matrices (full, dynamic, and static) was compared with the corresponding data matrices of the LH. To perform PCA on these matrices, first, the relative quaternions were linearized using the approach suggested in^26^ (we have also previously employed the same approach in^24,25^). A brief summary of the approach is as follows: A “column block” is a subset of the hand matrix and consists of the relative quaternions of a particular joint for all trials of all movements, hence it has a dimension of 86,400 × 4 (for the full set data matrix). There are 15 column blocks for the 15 joints. Markley’s algorithm is used to compute the mean of each column block^27^. The mean of a column block is then subtracted from each relative quaternion of that column block using quaternion conjugate multiplication, hence centering the column block about zero. Linearization of these column blocks is then performed using logarithmic maps (check equation S1 in supplementary material).

Upon linearizing the dataset using this approach, PCA was applied to the dataset which resulted in a matrix of eigenvectors (or synergies) and eigenvalues. Only the synergies that explained at least 95% of variance in the data were considered for further analysis. A simple search and match algorithm was used to match synergies between the LH and the RH for a participant^21,25^. Such a mapping is necessary because, previous studies have shown that when comparing the synergies obtained from different datasets, the synergies corresponding to higher eigen values map one to one (like synergies 1–4), whereas synergies corresponding to lower eigen values (like synergies 5–8) may not map one to one but could be invoked in different orders since these synergies lie close to each other in terms of explained variance^3^. Hence, for grouping similar synergies, different techniques like the greedy search method^21^, clustering method^29^, etc., have been previously employed.

To perform a search and match technique, the first synergy of RH was compared with all the synergies of the LH using the absolute value of Pearson’s correlation coefficient. The synergy pair with the maximum correlation coefficient was removed, and the next synergy of the RH was compared with the remaining synergies of the LH. This process was repeated until all synergies of the RH were paired with the corresponding synergies of the LH. From the matched pairs of synergies, top synergies that account for 90% variance in data were selected for comparison. This was done to ensure proper matching of higher-order synergies whose explained variances lie close to each other. The correlation coefficients of these synergy pairs were stored separately and plotted (See Fig. 5b for color map plot) for each participant. Furthermore, for each synergy pair, the corresponding correlation coefficients across participants were averaged, and the results are presented. In addition, the PC scores were compared between the RH and LH to strengthen and validate the results.

To visualize the synergies, eigen postures were plotted for the RH and the LH (See Fig. 6). In order to obtain the eigen postures, the following procedure was followed: The synergies obtained from the linearized data matrix were first converted to quaternions using exponential mapping (check equation S2 in supplementary material)^28^. Thus, for any given participant, the synergy matrix for the RH and LH had a size of 60 × 6. Here, the 6 columns represent 6 synergies. The 60 elements in every column encode the orientation information of 15 joints (15 joints × 4 valued quaternions) for the synergy. Each of the 4 valued quaternions along the column (in sequence) indicates the joint orientation of every joint. By entering the joint orientation information from a particular column in a 3D hand model, the synergy corresponding to that column can be visualized. The postures generated using this method are called eigen postures and provide information about what the synergies encode (i.e., which joints are being controlled by that synergy). However, it is straightaway not possible to visualize these synergies by posture reconstruction since the synergy values are zero-centered (this is because the mean had been subtracted initially while computing the correlation matrix). Hence, for proper visualization of the eigen postures, the base synergy postures were moved in either direction by adding and subtracting the mean value using quaternion conjugate multiplication (Eqs. (2) and (3)). The mean value for each joint of a particular synergy was computed across all participants and this was done earlier while generating the correlation matrix. This resulted in max and min eigen postures for each synergy. By this method, the synergies were visualized, as shown in Fig. 6.

2 $$Eigen\,posture_{max} = qs_{i} { } \otimes { }q\mu_{i}$$

3 $$Eigen\,posture_{min} = qs_{i}^{conj} { } \otimes { }q\mu_{i}$$

Here, $qs_{i}$ is the ith synergy, and $q\mu_{i}$ is the mean posture.

To further analyze the similarities in the synergies between the LH and RH, the ability of synergies of one hand to reconstruct the postures of the other hand was tested. For this purpose, a direct reconstruction and cross-reconstruction using the synergies that explain 90% variance in data was performed as follows: For direct reconstruction, the linearized data of the RH (i.e., the data just before application of PCA) was projected onto the synergies of the RH, and linearized data of the LH was projected onto the synergies of the LH. For cross-reconstruction, the linearized data of the RH was projected onto the synergies of the LH, and linearized data of the LH was projected onto the synergies of the RH. For cross-reconstruction, the new synergy order was used, which was obtained after performing the search and match operation. In other words, if synergies1,2,3,4,5 and 6 of the RH matched with the synergies 1,2,3,4,6,5 of the LH, the LH synergies were arranged as per the new order before reconstructing the RH data and vice versa. In the four cases just mentioned, since the linearized data was projected onto synergies which had a lower dimension, the dimension of the projected data was also reduced. This projected data (in synergy space) with reduced dimension was reconstructed back to the original full-dimensional measurement space and then converted to quaternions using exponential mapping (S2). This resulted in the following 4 reconstructed data matrices: RR (RH synergies, RH data projected), RL (RH synergies, LH data projected), LL (LH synergies, LH data projected), and LR (LH synergies, RH data projected). The RMSE was computed between the reconstructed data matrix and the original data matrix for all 4 conditions using (4) as suggested in^26^.

4 $$RMSE = { }\sqrt {\mathop \sum \limits_{i = 1}^{n} \frac{1}{n}\parallel\ln (Q_{1}^{conj} \otimes Q_{2} { })\parallel^{2}}$$

Here $Q_{1}$ is the reconstructed data (RR/LL/RL/LR) in quaternion format and $Q_{2}$ is the original data (RH data/LH data) in quaternion format.

To analyze the similarity in synergies between the RH and LH at the population level, the simple search and match algorithm that was previously applied between the LH and RH was applied again between all possible pairs of RH synergy matrices across participants. In brief, the RH synergy matrix of participant 1, consisting of top synergies (that explain 95% variance in the data), and the RH synergy matrix of participant 2 consisting of top synergies (that explain 95% variance in the data) was selected. The search and match algorithm was applied between the two matrices to give top synergy pairs corresponding to 90% variance in data and their corresponding correlation coefficients. The mean of these correlation coefficients was computed. This mean value is called the similarity index (SI). The SI was similarly computed for all possible combinations of RH synergy matrices across participants. The mean of all the SI values was then computed to get a single synergy similarity metric (SSM). Using the same computations mentioned in the preceding lines, the SSM for the LH was also obtained. An independent 2 sample t-test was used to test for differences in synergy at the population level.

## Results

### Dimensionality reduction using PCA

**Figure 4:**
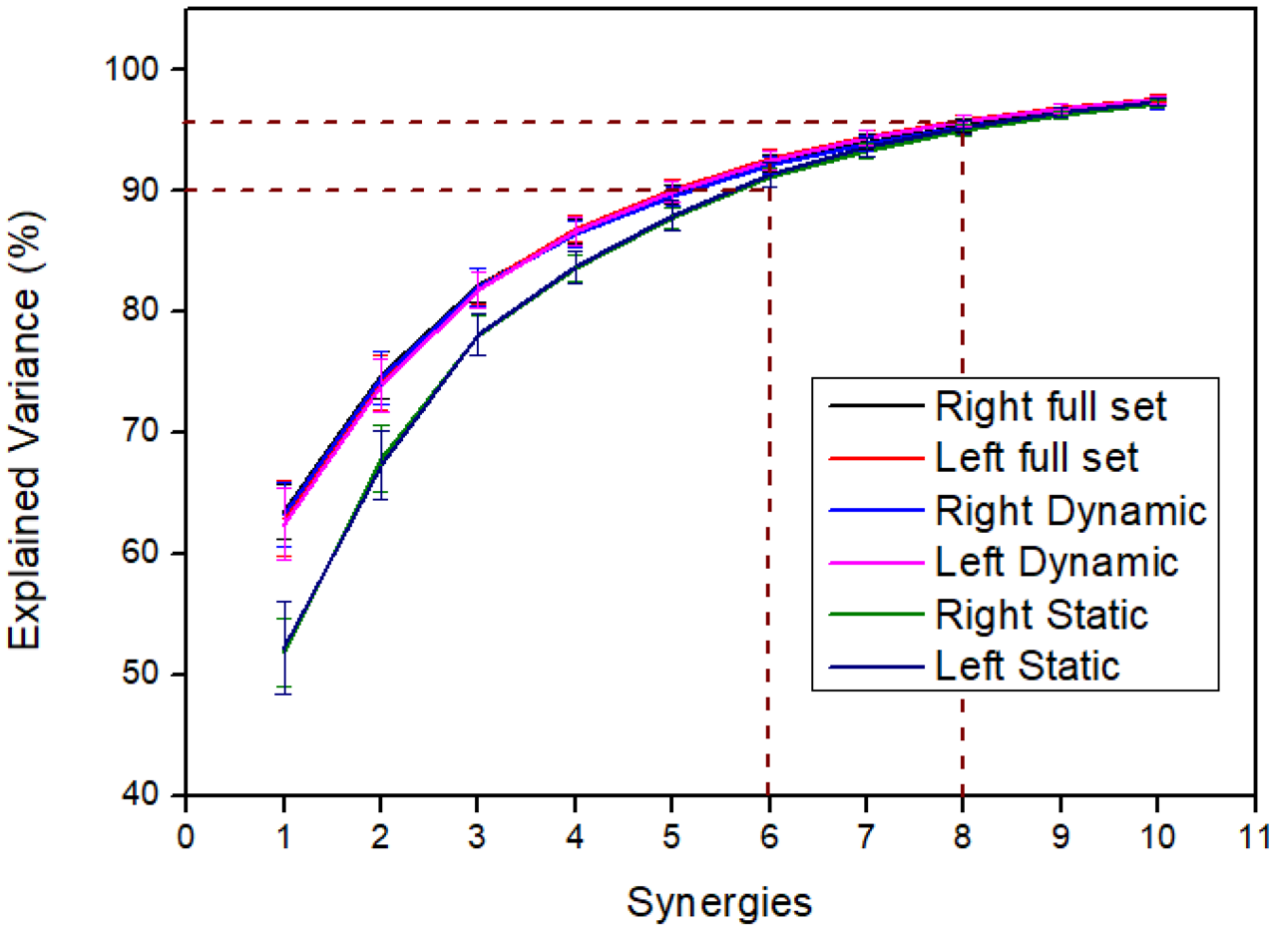
Scree plot for the LH and RH. The y axis indicates the percentage explained variance computed using the cumulative eigenvalues. The error bars indicate standard deviation (S.D). The first six synergies account for greater than 90% variance in data for all conditions – dynamic, static, and full set data. The first 8 synergies account for greater than 95% variance in data.

The eigen values from the PCA performed on the RH and LH dataset was used to obtain the scree plot for all three data sets (full, dynamic, and static) (See Fig. 4). From the plot, it can be seen that, for the full set and dynamic data, the first synergy explains greater than 60% variance in data for all the participants and the first three synergies explains greater than 80% variance in data. For the static data, the first synergy explains around 50% variance in data and the first three synergies explain greater than 75% variance in data. This is in accordance with the results of previous studies^1,3,5^. In this study the first eight synergies are selected for analysis as they explain greater than 95% variance in data and top 6 synergy pairs that explain 90% variance in data are utilized for the analysis.

**Figure 5:**
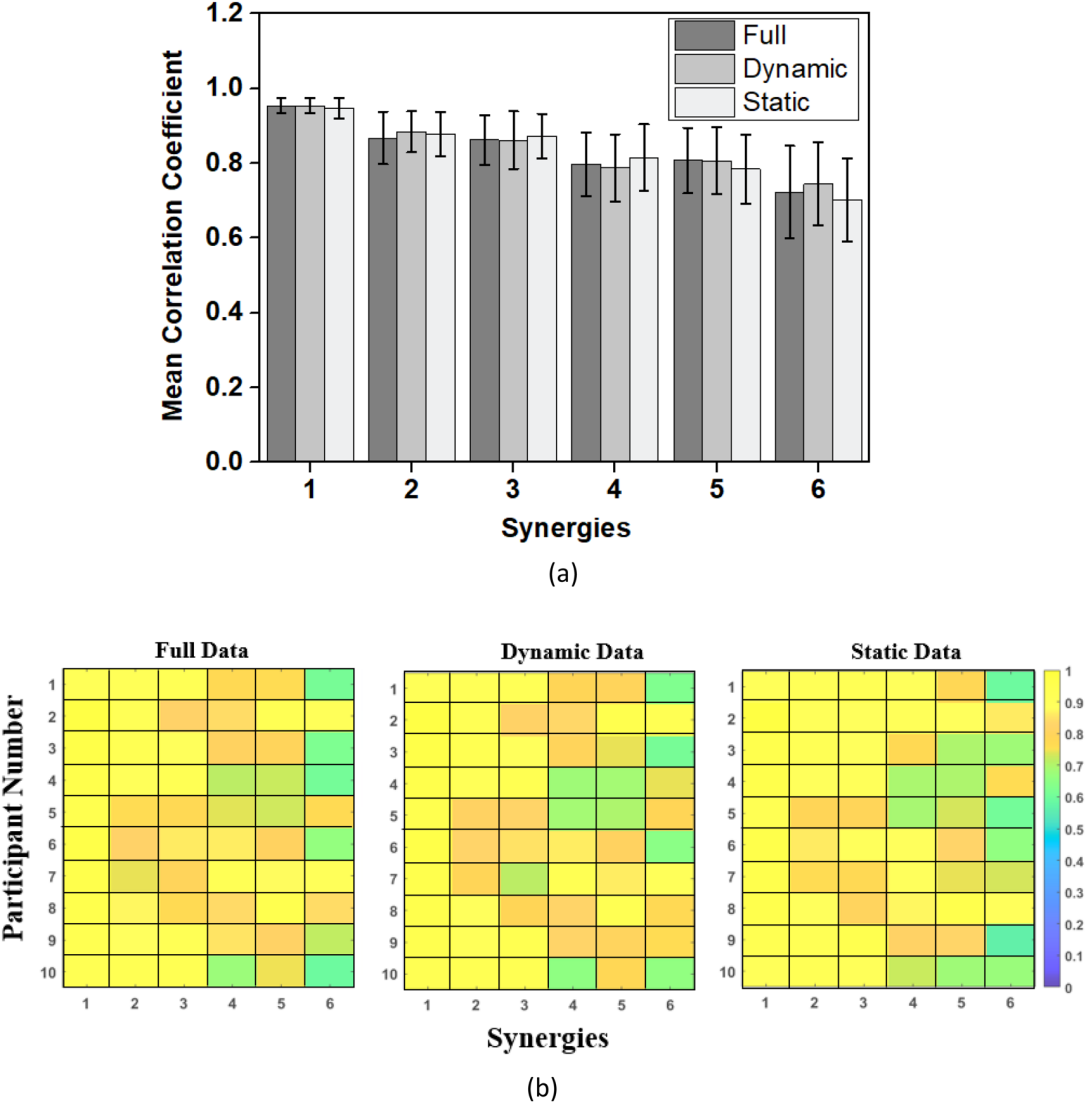
Comparison of the first six synergies between the LH and RH using Pearson’s correlation coefficient: (**a**) Mean CC for each synergy pair computed across all participants separately for the full set data, dynamic data, and static data. The error bars indicate standard deviation (S.D). (**b**) The CC between the RH and LH synergies are shown for each participant for the three datasets. Each value of the correlation coefficient was computed between the synergy of the RH and the corresponding matched synergy of the LH.

The mean of Pearson’s correlation coefficient (CC) across all participants, for each of the six synergy pairs (between a participant’s LH and RH) is plotted in Fig. 5a. The error bar indicates the standard deviation (S.D). From the plot, it can be seen that the correlation between the LH and RH synergies is the strongest for the first synergy (mean CC = 0.9541, 0.9540, and 0.9469 for the full set, dynamic and static data respectively). Synergies 2 to 5 are also strongly correlated having high mean CCs. The p values also support these results with the correlation (between the LH and RH synergies) being significant (p < 0.001) for the first 5 synergies. Among the six synergies, the sixth synergy has the lowest mean CC (CC = 0.7228, 0.7446, 0.7017 for full set, dynamic and static data respectively). Additionally, for the 6th synergy, the CCs across participants were relatively more variable when compared to the CC of other synergies (See Fig. 5) with the 3rd participant having the lowest CC of 0.6075 and 0.6121, for the full set and dynamic data. For the static data, the 9th participant showed a CC of 0.5712 for the 6th synergy pair. However, the p values for the 6th synergy for all the participants showed significant correlation (p < 0.02) for all 3 categories of data set (full, dynamic and static). A detailed breakup of the CCs for all the participants for the 1st six synergy pairs is presented in Fig. 5b. Since, the results for full set, dynamic and static data were similar, additional analysis and visualization is performed for the full set data.

**Figure 6:**
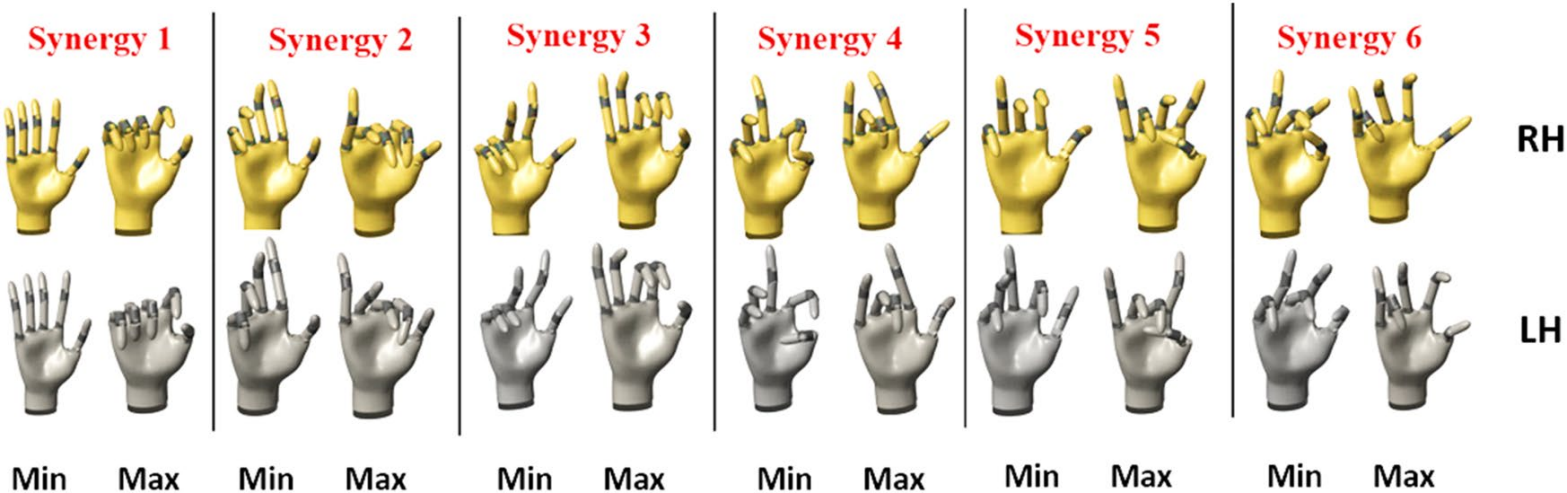
Eigen postures plotted for the first six synergies of participant 3 for the full set data. Many of the eigen postures of the LH and RH look very similar upon visual inspection.

To visualize the synergies, the eigen postures corresponding to the first 6 synergies computed from the full set data of participant 3 are shown in Fig. 6. This participant was chosen because the participant had the lowest CC value for the 6th synergy when compared to all the CC values across all the participants. Upon visually inspecting Fig. 5, it was observed that many of the eigen postures of the LH and RH were very similar. Furthermore, it was observed that the movements of the MCP and PIP joints were encoded in the first two synergies. This observation is in accordance with previous studies^1,12^. The higher order synergies are dependent on the postures used in the study.

**Figure 7:**
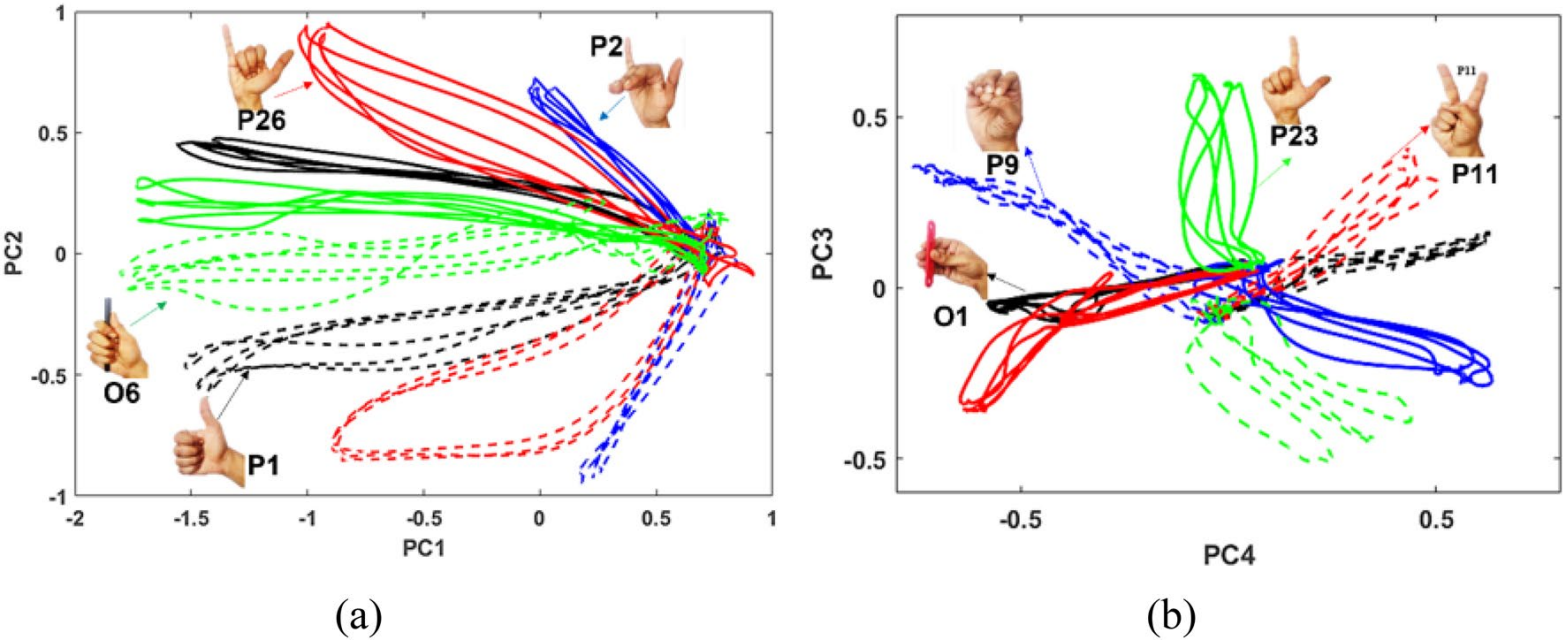
PC scores plotted for different postures. Solid lines indicate RH, and dashed lines indicate LH (**a**) PC1 and PC2 plotted for randomly selected postures for participant 7 (**b**) PC3 and PC4 scores for randomly selected postures for participant 1. The PC scores are plotted using the full set data.

In order to further analyze how the movements are encoded in the lower dimensional latent space for the LH and RH, the first 4 PC scores (PC1-4) were analyzed for different postures. Full set data was utilized for the visualization. The PC scores were analyzed by plotting PC1 versus PC2, and PC3 versus PC4, separately for randomly selected participants and movements. The PC1 vs PC2 plots for postures P1, P2, P26, and O6 for participant 7 are shown in Fig. 7a. Similarly, the PC4 vs PC3 score plots for postures P9, P11, P23, and O1 for participant 1 are shown in Fig. 7b. In the plots, the solid lines indicate RH, and the dashed lines indicate LH. As can be observed from the plots, the ranges of the PC scores for PC1 and PC2 are similar for the RH and LH except that they lie in different regions of the PC space. They lie in different regions for the following reason: upon computing the eigenvectors, the direction of the eigen vector can be along either direction of the line spanned by the eigenvector. This direction may be different every time the eigenvector is computed and is a computational artifact. Since the PC scores are plotted on a 2D surface by projecting the original data onto the space spanned by two eigenvectors, the location of the projection can be in any one of the four quadrants of the 2D space (the co-ordinate axis being the two orthogonal eigenvectors) based on the direction of the two eigenvectors. All the plots could have been plotted in the same quadrant by adjusting the directions of the eigenvectors, but this would have led to many overlapping plots. Hence, this was not done for the purposes of better visualization. PC3 and PC4 show slightly more dissimilarity in the ranges of the PC scores for the RH and LH postures/object grasps. This is in line with the correlation coefficients previously presented which showed that the correlation between RH and LH was lesser for PC4 (synergy 4) when compared to PC1. Thus, the PC scores provide further visual proof of the similarity of the lower dimensional representation of the RH and LH. Additional analysis of the PC scores for selected postures/object grasps are presented in the supplementary material.

**Figure 8:**
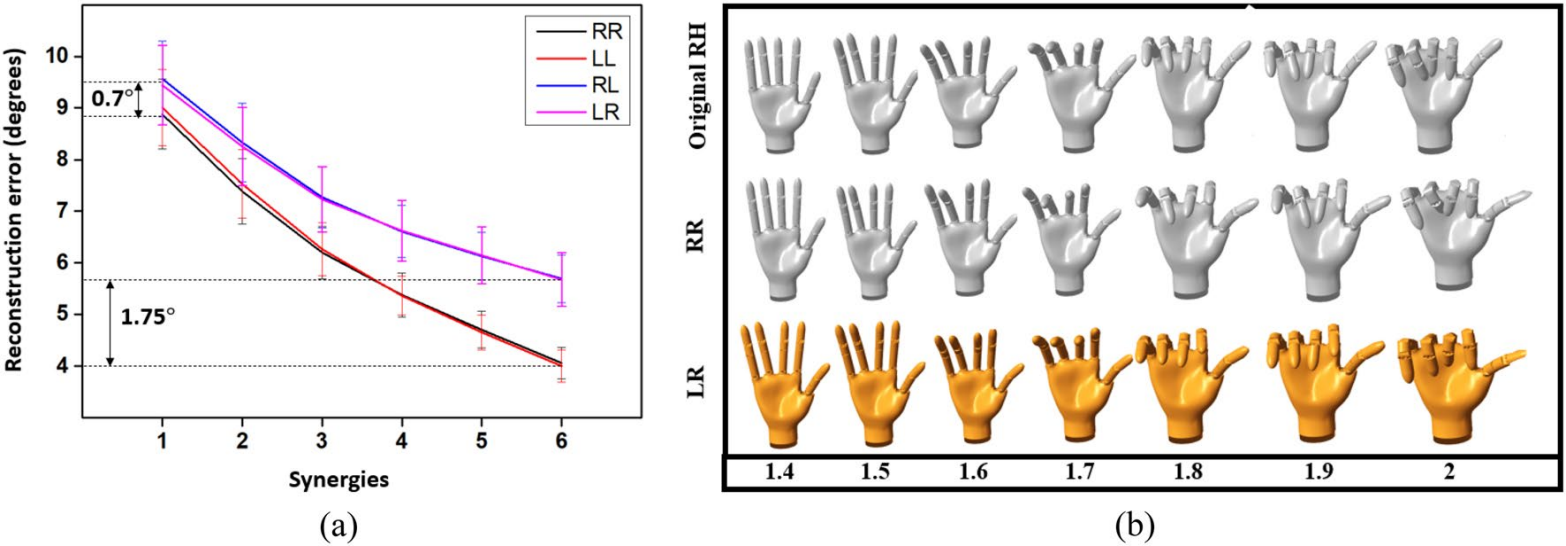
Reconstruction error for direct and cross-reconstruction. (**a**) Reconstruction error computed using RMSE for different combinations – RR, LL, RL, and LR. Minimum and maximum errors are shown on the plot for the 1st synergy and 6th synergy respectively. The plot is shown for the full set data (**b**) Comparison of original, direct reconstructed posture (RR) and cross reconstructed posture (LR) for a randomly selected posture of participant P7 using the first six synergies. The plot is shown for the first few seconds (from 1.4s to 2s), where the posture moves from a rest state to an intermediate active state. For cross-reconstruction, the new order of synergies obtained after the search and match operation was utilized.

The results of direct reconstruction and cross-reconstruction error for the full set data are shown in Fig. 8a. The plot represents mean reconstruction error across all participants, and the error bars indicate standard deviation (S.D). The reconstruction error was more for cross-reconstructions RL and LR than direct reconstructions RR and LL. The difference in mean reconstruction error between direct reconstruction and cross reconstruction varied between 0.75° to 1.75° (S1 to S6 respectively). Such low error values provide sufficient evidence that the synergies between the LH and RH are similar. A randomly selected posture for a participant P7 (who demonstrated slightly lower CC for synergy 2 and synergy 3 when compared to other participants) was selected to compare the reconstructed postures between RR and LR combination (Fig. 8b). This combination was selected to ensure that the same data was reconstructed from the RH and LH synergies, so as to visualize the differences if any, in the synergies only and not reflect any minor changes in the original postures. The postures were reconstructed using all six synergies. As can be seen from the figure, visually, the differences between the original, direct reconstructed, and cross-reconstructed postures are very little.

**Figure 9:**
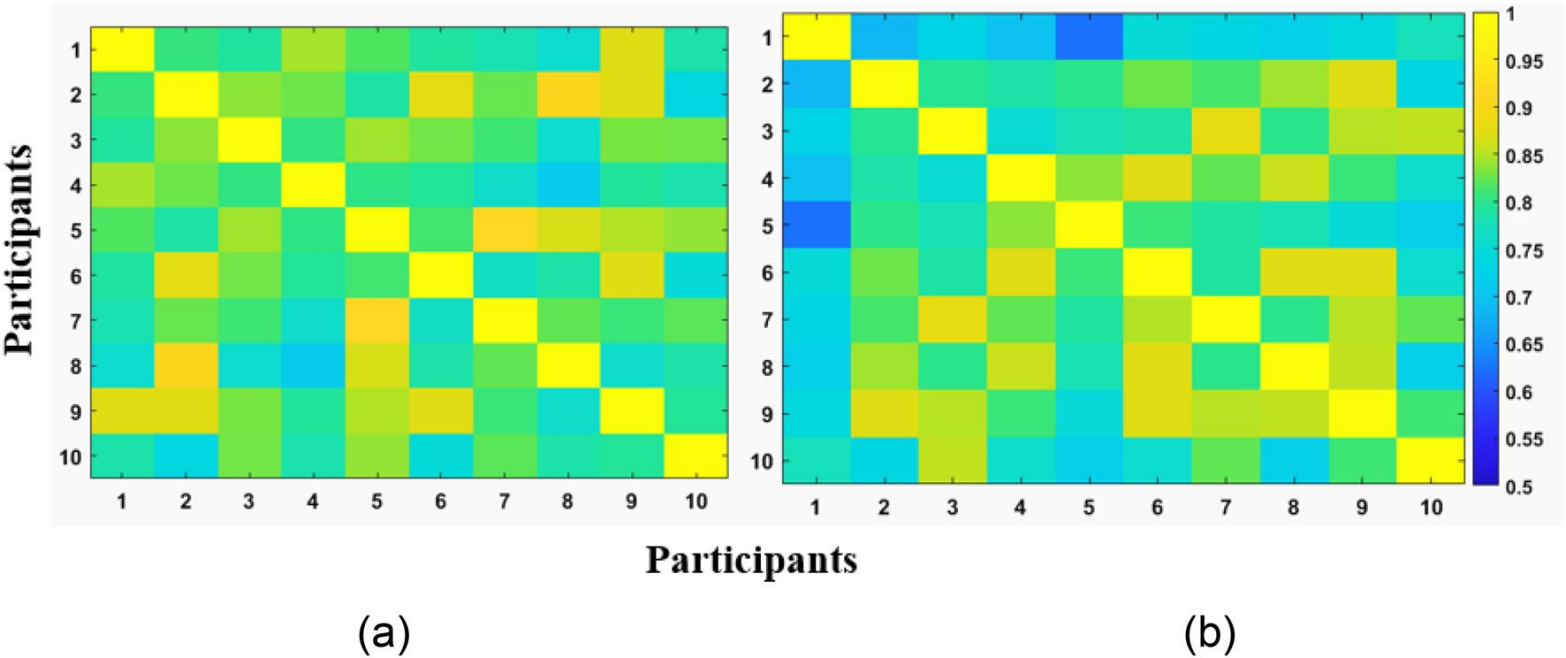
Comparison of synergies at the population level. CC’s are computed for all possible synergy pairs of (**a**) RH of all participants and (**b**) LH of all participants. The plots are shown for the full set data. The CCs were computed after rearranging the synergies based on a search-and-match technique.

Finally, for comparison between the RH and LH synergies at the population level, SI was computed for the RH and LH for all the participants for the full set data. The color map plot of the computed SSI’s for the RH and LH are presented in Figs. 9a and b respectively. Finally, the SSM was obtained by computing the mean of the SI for the RH and LH. The SSM for the RH and LH are 0.79 ± 0.058 (mean ± S.D) and 0.81 ± 0.043 respectively. An independent sample t-test revealed no significant differences between the population means (p = 0.0785) for a significance level of 0.05.

## Discussion

In this study, we examined if synergies of the DOM and NDOM hand are different in right-handed participants. We hypothesized that the synergy-based commands generated from the left and right hemispheres to control the contralateral right and left hands, respectively, were different. This hypothesis was backed by various studies that have found neural correlates for hand synergies and various studies that have provided evidence for structural and functional differences between the motor areas of the left and right hemispheres. While differences in control between DOM and NDOM upper limbs have been documented through the dynamic dominance hypothesis^22,23^, studies that support such a difference for the kinematics of distal segments like the fingers are scarce.

To test our hypothesis, we conducted an experiment where ten participants performed 36 hand movements involving ASL postures, Bharatanatyam postures, and object grasps with the RH and LH in two separate sessions. Analysis of synergies of the RH and LH using PCA and comparison of CCs between the synergies of the RH and LH revealed no significant differences between the synergies of the two hands. To investigate this further, two additional analysis was performed. Firstly, the variation of hand kinematics in the lower dimensional latent space was visualized by plotting the PC scores. Secondly, the ability of synergies of one hand to reconstruct the postures of the other hand through cross-reconstruction was demonstrated by generating hand postures. PC score plots visually showed similarity between the RH and LH movement data in the lower dimensional latent space. Cross-reconstruction analysis showed that the postures obtained through cross-reconstruction and direct reconstruction were comparable. Finally, a comparison of synergies was performed at the population level, wherein the similarity of synergies across all RH pairs of participants was compared with that of the LH. Even at the population level, the synergies of the RH and LH were comparable.

Studies focusing on the comparison of synergy patterns of the RH and LH are scarce, hence limiting the comparison of our study to only a few studies. Here, we compare the current study with two previous studies, one on postural synergies by Jarasse and colleagues^5^ and the other on muscular synergies of the RH and LH by Liang and colleagues^21^. The study by Jarasse et al., compared the postural synergies of the RH and LH during object grasps. They found significant differences in the PC scores of the 1st and 4th synergies between the RH and LH^5^. There are some differences in the method of analysis between the current study and the study by Jarasse et al. Firstly, Jarasse et al., performed a vertical concatenation of the data from all participants before analysis. However, a recent study that analyzed the sharing of synergies across participants demonstrated that analyzing synergies of all participants together results in merged unreal synergies^29^. Hence, these unreal synergies might not give insights into the actual control strategies employed by the CNS. Secondly, only static hand postures were analyzed by Jarasse et al., whereas in the current study, static data, dynamic data and data from the entire trial, which included the combined data of static and dynamic movements were separately analyzed. Analyzing data from the entire trial would give better insights as the data would contain dissimilarities, if any, in the movements of the RH and LH. These dissimilarities might contain information about differences in the control strategies employed by the two cerebral hemispheres in controlling movements of the contralateral hands. Finally, in Jarasse et al.’s study, only the PC scores were compared between the hands and not the synergy patterns. In the current study, the synergy patterns were compared for the main analysis, and the PC scores were also compared for visualization. It is difficult to present the results of the PC analysis in a concise manner since this study has 36 postures/object grasps. Hence, the results of the PC analysis are presented in the supplementary material. The results of our analysis showed that the synergies of the RH and LH are not different. This result is consistent with a recent study by Liang and colleagues, that compared the muscular synergies of the RH and LH^21^. Their study revealed that the synergies were not different between limbs at the individual level as well as at the population level for right handers. The method of analysis in the current study is similar to that of Liang’s study.

Many synergy-based studies in the literature have used simple postures and object grasps as part of their experiment^1,5,6,21,30,31^. In accordance with these studies, the current study also used simple postures and object grasps (36 in total). Contrary to our hypothesis, the results showed that the LH and RH synergies were not different. This result suggests that in spite of the structural and functional differences between the motor areas of the two cerebral hemispheres, from a kinematics perspective, the control strategies employed by the DOM and NDOM hemispheres are not different. A possible explanation for this is as follows: Studies have provided evidence that the right hemisphere's control of the LH is significantly influenced by the transcallosal projections from the dominant left hemisphere^32,33^. For e.g., Sperry and colleagues reported that for two right-handed patients whose corpus callosum had been surgically transected to treat epilepsy, the voluntary control of the left hand was difficult in the initial months following the surgery^32,33^. This suggests the importance of the left hemisphere in the control of movements of both hands. Hence, instead of two separate control strategies being lateralized to the DOM and NDOM hemispheres, respectively, there probably exists a single control strategy that is used by both hemispheres.

However, we believe that the results of the current study cannot be conclusive for the following reason: If we consider some common everyday bi-manual tasks that involve intricate movements of the fingers, such as opening a bottle, opening a lock, passing a thread through the eye of a needle, turning screws using a screwdriver, using scissors to cut a paper, sharpening a pencil, etc., the DOM hand is consistently used for the manipulative part of the task and the NDOM hand is consistently used for providing stability. This is in line with the dynamic dominance hypothesis that was documented for arm movements^22,23^. This can be interpreted as the DOM hemisphere being better at generating commands for the manipulative part of the task and the NDOM hemisphere being better at generating commands for the stability part of the task. In this regard, we speculate that the DOM and NDOM hemispheres use different control strategies, which manifests as them being better at generating certain types of commands. Now, if we instruct the NDOM hand, against its natural choice, to perform the manipulative part of the task, and if the NDOM hemisphere uses a different control strategy than the DOM hand to perform the same task, then this should be reflected in the kinematic data. Keeping these points in mind, we claim that kinematic data from simple movements (as commonly used in synergy studies) alone will not contain sufficient information to conclusively test our hypothesis. Apart from this, many of the postures/object grasps used in the current study are new to the participants, i.e., the participants are not well trained for these tasks both in the neural space and the synergy space. The results of the current study show that the control strategies employed by the CNS for these new/unfamiliar tasks are the same for both the DOM and NDOM hands. Perhaps learning or training for a task could lead to the generation of new synergies. It would be interesting to see whether conducting the same experiment for tasks in which one of the hands is well-trained when compared to the other hand would result in differences in synergies between the DOM and NDOM hands. Maybe using activities of daily living (some of which are mentioned earlier in this paragraph) in which one hand is well-trained in the synergy space as well as the neural space when compared to the other hand could further help in teasing out the differences in the synergies between the DOM and NDOM hands if any.

In order to further validate the results of the current study, we propose performing synergy analysis on kinematic data of more complicated, well-trained bi-manual movements such as those mentioned in the preceding paragraph. Such an experiment could involve two sessions, one in which the DOM hand does the manipulative part of the task and the other in which the NDOM hand does the manipulative part of the same task. Now, the data set obtained from the two sessions could be compared. If the DOM and NDOM hemispheres indeed employ a single control strategy to control the hands, then the DOM and NDOM hand synergies should not be different for such a study.

It is also possible that an analysis at the level of muscle synergies (which is closer to the CNS space) during bimanual manipulations could better elucidate the differences in the control of the DOM and NDOM hand due to the implications of force control involved in complex tasks. A wide variety of tasks must be utilized, spanning a large range of hand uses that could represent the capabilities of the CNS. Additionally, analysis of fingertip force coordination in complex tasks like that reported in^36^ could be performed for the NDOM hand, and juxtaposing its performance with the DOM hand could reveal differences if any between the DOM and the NDOM hands.

Finally, from a translational point of view, previous studies have hypothesized that people with neuromotor disorders could have a few synergies missing compared to that of the healthy population^34,35^. The similarity in synergies observed between the hands while performing simple hand postures and object grasps could be utilized to identify missing synergies between hands in people with neuromotor disorders like stroke. In such cases, the synergies of the more affected hand could be compared to the synergies of the lesser affected hand of the same person to gauge rehabilitation performance and enhance treatment outcomes.

## Concluding comments

In this study, the RH and LH synergies were compared using kinematic data of simple hand movements. We hypothesized that synergies of the RH and LH would be different. This was because we speculated that the two hemispheres employed different control strategies owing to asymmetries in the structure and function of their respective motor areas. A comparison of synergies obtained through PCA was made both at the individual level as well as at the population level. Contrary to our hypothesis, the results showed no difference in synergies between the RH and LH at both levels. Additional analysis through visualization of PC scores and computation of cross-reconstruction errors further strengthened the obtained result. This result indicates that the DOM and NDOM hemispheres use similar control strategies to control the contralateral hands. However, we claim that this conclusion is not definitive and needs to be further investigated using kinematic and muscle EMG data from more complicated bi-manual tasks involving intricate movements of the fingers.

### Supplementary Information

Supplementary Information.

## Data Availability

The data collected for this study is available upon request by contact with the corresponding author.
