# Supplementary material for: Comparison of synergy patterns between the right and left hand while performing postures and object grasps
Source: Sci Rep. 2023 Nov 20;13:20290. doi: 10.1038/s41598-023-47620-9 (PMC10662439; doi:10.1038/s41598-023-47620-9)
Supplement: Supplementary file 1 — Supplementary Information. [file 41598_2023_47620_MOESM1_ESM.docx]

1. **Logarithmic mapping and Exponential mapping**

Direct application of linear operations like PCA on quaternions is not recommended since quaternions are defined on a nonlinear domain. For this purpose, the quaternions are first mapped to a linear domain using logarithmic mapping, PCA is applied and then the linearized orientations are converted back to quaternions [26].

For linearizing a quaternion, the quaternion of the from q= (qw, qv) was mapped to a 3D plane using the logarithmic mapping (S1). The new element in the 3D plane will be a 3D tuple v= [$v_{x}, v_{y}, v_{z}]. This$element v encodes information of both axis and the corresponding angle.

$v=ln q= \left( ln(\left| q \right|, \left( \frac{1}{\left\| q_{v} \right\|} arcos \frac{q_{w}}{\left| q \right|} \right)q_{v} \right)$ (S1)

After applying linear operations on v, the quaternion q is obtained from its linearized form using exponential mapping (S2)

$q=e^{v}$=$\left[ cos\left( \frac{\theta}{2} \right), sin\left( \frac{\theta}{2} \right)\frac{v}{\left\| v \right\|} \right]$ (S2)

where $\theta=\left\| v \right\|$

While converting the orientation in linearized form (v) back to the corresponding quaternion (q), a singularity could occur if v has crossed $\pi$ radians. Since in our application, only relative orientations were used and none of the relative orientations finger phalanges go beyond $\pi$ radians, the mapping from v to q is singularity free. Details on singularities during such a conversion can be found in [28]. The Matlab functions ‘quatlog’ for logarithmic maps and ‘quatexp’ for exponential maps were utilized in this study.

1. **Visualization and comparison of PC scores of DOM and NDOM hands for various postures and object grasps**

Comparison of the PC scores was performed for 20 postures/object grasps ( five in each section where the division of the sections is as follows – Section 1: Bharatnatyam postures (P1-P9), Section 2: ASL numbers (P10-P19), Section 3: ASL letters (P20-P26) and Section 4: Objects(O1-O10).

Only five postures/object grasps were selected per section for the analysis, and only the first four PCs per posture/object grasp were visualized for the following reasons:

1. PC scores are dependent on postures and must be analyzed for each posture separately and for all PCs. In this study, since there are 36 postures, it would be difficult to present the result for all the postures and all the PCs per posture concisely.
2. While visualizing the scores, the scores could overlap, making it difficult to visualize. Hence five postures were selected per section whose first four PC scores showed some demarcation amongst themselves during visualization.

The PC scores of the selected postures/object grasps are plotted in Fig S1. The following procedure was followed for the analysis.

1. The PC score of PC1 of a posture/object grasp was averaged across the 3 trials for a participant.
2. This averaged PC score was then averaged across all participants.
3. This was repeated for the remaining PCs (PC2-PC4). Only 4 PCs were selected for brevity.
4. The average and S.E.M of the 4 PCs for the posture/object grasp were plotted.
5. This procedure was repeated for all the selected postures/object grasps.

Visual inspection of the plots in Fig S1 indicates that the PC score variation for all 4 PCs between the DOM and NDOM hands are not different.


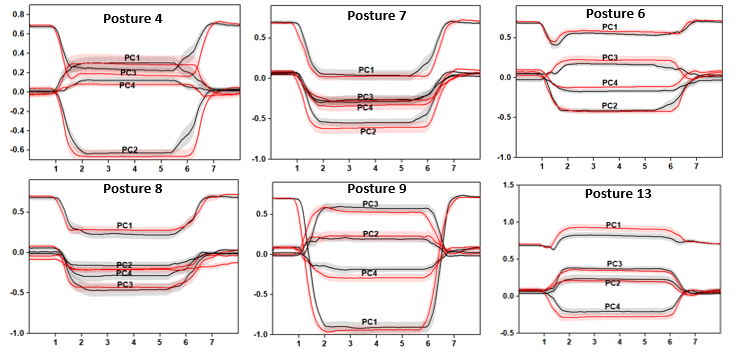


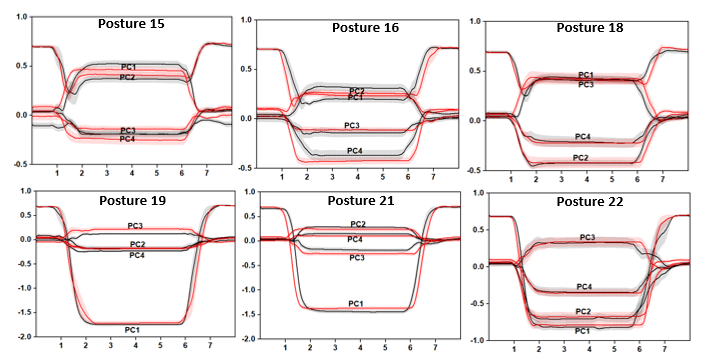


**Time (seconds)**

**PC scores**


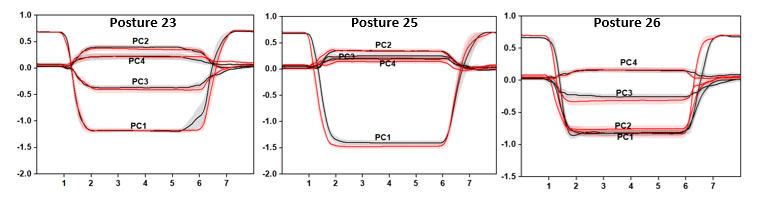


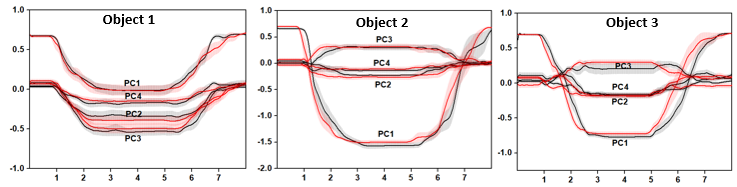


**PC scores**


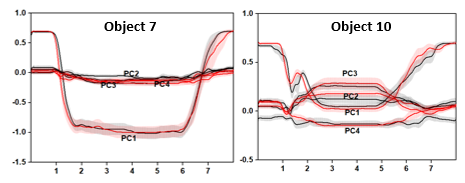


**Time (seconds)**

**Figure S1.** Comparison of PC scores between the DOM and NDOM hands for the first 4 PCs. Black lines and red lines indicate the average PC scores for the DOM and NDOM hands, respectively. The shaded area indicates the S.E.M.
